# Visual, Musculoskeletal, and Balance Complaints in AMD: A Follow-Up Study

**DOI:** 10.1155/2016/2707102

**Published:** 2016-10-18

**Authors:** Christina Zetterlund, Hans Olof Richter, Lars-Olov Lundqvist

**Affiliations:** ^1^The Low Vision Centre, Region Örebro County, Örebro, Sweden; ^2^University Health Care Research Center, Faculty of Medicine and Health, Örebro University, Örebro, Sweden; ^3^School of Health and Medical Sciences, Örebro University, Örebro, Sweden; ^4^Centre for Musculoskeletal Research, Department of Occupational and Public Health Sciences, Faculty of Health and Occupational Studies, University of Gävle, Gävle, Sweden

## Abstract

*Purpose.* To investigate whether patients with age-related macular degeneration (AMD) run a potentially higher risk of developing visual, musculoskeletal, and balance complaints than age-matched controls with normal vision.* Methods.* Visual assessments, self-rated visual function, self-rated visual, musculoskeletal, and balance complaints, and perceived general health were obtained in 37 AMD patients and 18 controls, at baseline and after an average of 3.8 years later.* Results.* At follow-up both groups reported decreased visual acuity (VA) and visual function, but only AMD patients reported significantly increased visual, musculoskeletal, and balance complaints. Decreased VA, need for larger font size when reading, need for larger magnification, and decreased self-rated visual function were identified as risk markers for increased complaints in AMD patients. These complaints were also identified as risk markers for decreased health. For controls, decreased VA and self-reported visual function were associated with increased visual and balance complaints.* Conclusions.* Visual deterioration was a risk marker for increased visual, musculoskeletal, balance, and health complaints in AMD patients. Specifically, magnifying visual aids, such as CCTV, were a risk marker for increased complaints in AMD patients. This calls for early and coordinated actions to treat and prevent visual, musculoskeletal, balance, and health complaints in AMD patients.

## 1. Introduction

Age-related macular degeneration (AMD) is one of the most common reasons of visual impairment in the western world [1, 2]. The prevalence of AMD is approximately 2% at the age of 40, with an increase to about 17%–35% at the age of 80 [3, 4]. Patients with AMD experience irreversible and progressive visual loss, leading to reduced quality of life [5–7], reduced health, and increased risk of mortality [8, 9]. In step with visual deterioration AMD patients report additional complaints, such as loss of postural stability [10–13], fall accidents [14], and neck/scapular area complaints [15–17]. In primary health care these symptoms are often downplayed as part of the aging processes [18] and not typically associated with visual impairments [6, 19]. However, research indicates that people suffering from AMD may pose a higher risk of developing visual, musculoskeletal, and balance complaints [20].

Aging alone does not predict the development of AMD. Genetics, lifestyle, body mass index, and smoking have all been found to increase the risk of developing AMD [2, 3]. AMD is identified by retinal changes beyond what are age-normal [1, 3, 18]. Early stages are associated with minor visual disturbance, followed by increased retinal changes during the intermediate stages and the development of pronounced visual deterioration at the late stages [1, 2]. AMD follows no particular schedule, but studies show that approximately 20% of cases escalate from intermediate to late stage within 6 years [1, 2, 21].

As vision deteriorates the visual system is subjected to increased levels of strain. At some point this may adversely affect visual performance and further distort visual feedback to other systems that are normally supported by visual inputs such as the musculoskeletal and the postural systems. As a consequence, AMD patients will need new strategies to cope with everyday activities. There are a number of enhancing devices to help AMD patients adjust to their deteriorated vision. Although magnifying aids are aimed at facilitating everyday life, they come with some drawbacks such as reduced field of view or the need to use both hands or adopt awkward postures [22, 23]. Straining conditions such as orbicularis squinting (to increase effective focus imaging) or unfavorable gaze-angles (to locate the best viewing visual field or facilitate convergence movements) may also occur as the person struggles to acquire an acceptable image, which may in turn impose increased strain in the muscles used for positioning the head [16, 17, 24, 25]. Visual deterioration also negatively influences postural control as this is largely based on adequate visual feedback [12–14]. Hence, visual deterioration and increased use of visual aids may increase the risk of musculoskeletal and balance complaints [12, 13, 15–17, 26]. However, our knowledge of what influence visual loss in AMD may pose on musculoskeletal and balance complaints is limited.

Previous research addressing the impact of visual loss in AMD patients on visual, musculoskeletal, and balance complaints is limited or has used cross-sectional design [20]. This research has identified concurrent relationships; however, less is known about the influences of visual decline in AMD on visual, musculoskeletal, and balance complaints across time. By using a longitudinal cohort design these issues can be addressed. Therefore studies using longitudinal design are warranted in order to investigate to what extent visual deterioration influences visual, musculoskeletal, and balance complaints. The present study aims to identify risk markers for increased visual, musculoskeletal, and balance complaints and perceived general health in patients with AMD by using a longitudinal cohort design. Based on the previous research described above, we hypothesized that visual, musculoskeletal, and balance complaints should

- increase more in AMD patients than in age-matched individuals with age appropriate vision,
- increase with increased visual deterioration,
- negatively affect perceived general health.

## 2. Methods

### 2.1. Design

This study has a prospective longitudinal case/control design, in which a group of AMD patients and an age- and sex-matched reference group with normal vision were assessed twice.

### 2.2. Participant Selection

At baseline, AMD patients were recruited in consecutive order from the queue system of the Low Vision Centre at Region Örebro County, Sweden. Inclusion criteria were being diagnosed with late- or intermediate-stage AMD according to an ophthalmologic examination at a hospital eye clinic with best corrected visual acuity (BCVA) worse than 0.5 logMAR and no additional eye disease. The patient should have had the AMD diagnosis for at least one year in order to adapt to the visual impairment and become accustomed with use of magnifying visual aids.

Individuals in the reference group were recruited from relatives and companions of the AMD patients visiting the clinic. Inclusion criteria included age-normal BCVA defined as better than 0.10 logMAR with correction if needed and without any known eye disease.

Actual refraction and BCVA were tested in both groups in an eye-examination conducted by an optometrist at the low vision clinic. Individuals in either group were excluded at baseline or at follow-up if they were medically diagnosed with musculoskeletal or balance disorders, such as whiplash, arthritis, myalgia, or Parkinson's disease.

### 2.3. Participants

Descriptive data are shown in Table 1. At baseline in 2008/2009 the study included 88 individuals, 64 cases (43 women/21 men, mean age 78.6 years, SD = 5.81, and range 61.8–85.9 years), and 24 referents (11 women/13 men, mean age 73.9 years, SD = 6.08, and range 64.9–83.0 years). In 2012, a mean of 3.8 years and SD = 0.46 years later, the former participants were contacted by telephone to schedule the follow-up appointment. At follow-up 55 individuals remained, including 37 cases (28 women/9 men, mean age 81.1, SD = 5.43, and range 67.2–89.2 years) and 18 referents (11 women/7 men, mean age 77.6, SD = 5.60, and range 69.0–78.1 years).

None of the AMD patients were diagnosed with wet neovascular AMD at baseline; however, six AMD patients have had ranibizumab injections prior to baseline. Twenty AMD patients (31%) had reached “late AMD” at baseline according to clinical classification of age-related macular degeneration [1]. Two participants (one AMD patient and one referent) have had monocular cataract surgery with a replaced intraocular lens.

**Figure 1:**
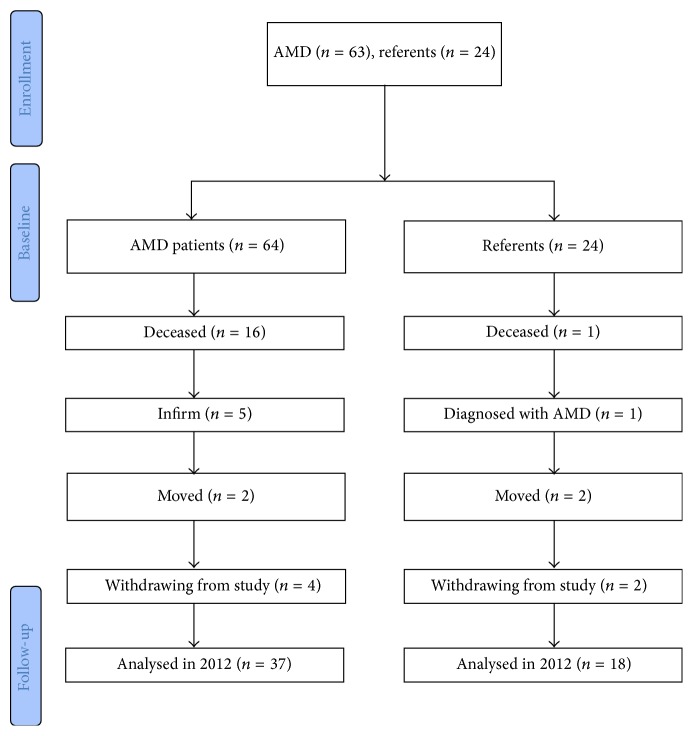
Consort chart showing number of allocated cases and controls at baseline and motives for not participating in follow-up approximately 3.8 years later.

Among the 27 cases not participating in the follow-up 16 were diseased, 5 were infirm or suffered from dementia and were not able to participate, 2 had moved, and 4 declined without giving any specific reason. Among the 6 referents not participating in the follow-up, one was deceased, one had moved, one had acquired AMD, and 3 declined without giving any specific reason. Consort flow chart is shown in Figure 1.

The study was performed according to the tenets of the Helsinki Declaration. The Regional Ethical Review Board in Uppsala, Sweden, approved the study. Informed consent was collected from all participants at baseline and at follow-up.

### 2.4. Visual and Optometric Assessments

*Best corrected visual acuity* (BCVA) using habitual visual aids (ordinary spectacles or contact lenses) was assessed under monocular and binocular viewing conditions using the Early Treatment Diabetic Retinopathy Study (ETDRS) test chart [25]. If VA was very low, the Bailey-Lovie letter-by-letter chart was used to capture VA beyond 1.0 logMAR [27].

*Critical print size *was assessed by the smallest font size that could be read fluently/best acceptable reading pace, with the use of the participants' normal visual aids. CPS was measured in points (p), where 1 p = 1/72 of an inch. A font size of 8 p is commonly used in newspapers and is equivalent to a Snellen notation of N8 or 1M. CPS was assessed as participants read the appropriate printed texts, ranging from 4 p to 64 p.

*Reading distance* was assessed by measuring the distance in cm between the text and the eyes when the participant was reading the near charts with assistance of their normal visual aids.

*Visual aids* were those the participants normally used when reading (if any). Visual aids contained head-worn visual aids (single vision reading glasses, bifocals, or progressives) used alone or combined with handheld magnifiers or closed circuit television (CCTV).

The* magnification *used while reading was estimated by summing up the dioptric power (D) of those visual aids that normally were used simultaneously. The amount of dioptres was then transformed into units of magnification by dividing the sum of D by 4 based on a simplified commonly used nominal magnification transformation formula (*M* =* F*/4). This calculation does not give a perfect value of the provided enlargement [23, 27, 28], as it is not based on the equivalent viewing distance. However, it gives a sufficient estimate of graduating magnitude of visual aids. For example, if reading glasses of +8 D were used, this refers to a magnification of 8/4 = 2x, but combined with a handheld magnifying aid of +20 D, this refers to a magnification of 8/4 + 20/4 = 7x. This assumes a reading distance of less than 20 cm, whereof at least half the distance refers to the distance between the specs and the magnifying aid. In clinical practice, most of the elderly have difficulties accomplishing a shorter reading distance than what is provided by reading glasses above +8.0, which is the reason why the additive handheld magnifying aid is often combined with their best reading glasses to overbridge effects from the continuous visual deterioration and does not shorten the reading distance any further [22, 23]. Controls also used near visual aids, in order to compensate for the distance, which was estimated in the same manner.

### 2.5. Self-Rated Assessments

*Near visual function *was assessed on the Near Activities Subscale of the National Eye Institute-Visual Function Questionnaire 25 (NEI-VFQ 25) [29]. The Visual Function Questionnaire-Near Activities Subscale (VFQ-NAS) consists of six questions and has shown excellent internal consistency and reliability (Cronbach alpha 0.91) as well as convergent validity with BCVA and health-related quality of life among patients with AMD [30, 31]. Each question is answered by using one out of six available alternatives. The first five alternatives describe the quality of visual function, ranging from 0 to 100 at equal steps (i.e., 0, 25, 50, 75, and 100). The sixth alternative, “stopped doing this for other reasons or not interested in doing this,” is not related to the quality of visual function and therefore does not contribute to the total VFQ-NAS score. The sum score from the six questions was divided by the number of contributing questions to form a total near activity visual function score. Generally, a total score above 80 indicates minor visual problems and a score of 70 or less is considered clinically significant.

*Visual, musculoskeletal, and balance complaints* were measured on the visual, musculoskeletal, and balance complaints questionnaire (VMB). The VMB has adequate psychometric properties and convergent validity in people with visual impairments [32]. It consists of 15 questions, with five questions each in visual (VMB-V), musculoskeletal (VMB-M), and balance (VMB-B) domains. The questions are rated on visual analogue scales, ranging from 0 (no problem at all) to 10 (problems all the time), with verbal anchors at 3 (occasionally) and 7 (quite often). The sum of the five scores in each domain is then calculated, with scores ranging from 0 to 50. The validated VMB-scale has been revised, with some minor changes [32]. The present study was performed with the use of the original VMB-scale [20], which was used at baseline and then at follow-up allowing for detection of individual changes using GEE.

*General health *was assessed in a yes/no format by a single question. The participant could choose from two alternatives, feeling healthy (1) or not feeling healthy (0).

### 2.6. Statistical Analyses

Data analyses were performed using IBM SPSS Statistics version 22 (IBM Corp, Armonk, NY). Descriptive statistics were used to summarize participant characteristics. Most of the data were positively skewed, which does not necessarily indicate a problem with the scales but reflects the underlying nature of the construct in focus. Therefore, nonparametric tests were used on the data. Wilcoxon signed rank test was used to compare differences across time and Mann–Whitney* U* test was used to compare differences between groups. Generalized estimation equation (GEE) was used to obtain robust parameter estimates and standard errors and to further estimate the correlation of multiple observations for each subject over time [33]. The GEE is an extension of generalized linear models, which facilitate regression analyses of dependent variables that are not normally distributed. By using marginal models the analysis gives an average response for observations sharing the same covariates as a function of the covariates; that is, for every one-unit increase in a covariate across the population in focus, GEE tells the user how much the average response should change; GEE can thus account for correlations in repeated measures, and the interpretation of the estimates is much the same as that in ordinary least squares regression when the dependent variable is normally distributed. In the present study, each predictor variable was regressed separately on each of the VMB to identify significant risk markers for visual, musculoskeletal, and balance complaints. An unstructured working correlation matrix was used. Since the VMB variables were positively skewed, the GEE regression models were specified with a log link function. The influences of predictor variables on participants' perceived health (dichotomous data) were evaluated using GEE logistic models, which were expressed in odds ratios (OR) and 95% confidence intervals (95% CI). A *p* value < 0.05 was regarded as significant.

## 3. Results

At baseline there was no significant difference in sex ratios between cases and referents, but there was a trend for a difference at follow-up (*χ* ^2^ = 5.33, *p* = 0.069). This difference was mainly due to a larger number of dropouts among men with AMD (57%) than among women with AMD (35%) at follow-up. There was also a difference in age between groups at baseline (*U* = 445, *p* = 0.002) but not at follow-up (*U* = 256, *p* = 0.10).

As shown in Table 1, cases had worse BCVA at baseline than referents, needed larger font size when reading, used shorter reading distances, and needed greater magnification. Fewer cases than referents used progressives, but several more of them used a combination of different near visual aids. Cases also reported lowered near visual function, lowered perceived general health, and more visual and balance complaints, but not significantly more musculoskeletal complaints than referents.

At follow-up, within group analyses revealed that both groups showed deteriorated BCVA and visual function (Table 2). Cases needed larger font size when reading and more magnification, but referents did not. However, referents needed significantly shorter reading distances at follow-up, which was not found for cases, although cases still needed shorter reading distances than referents (*U* = 49.5, *p* < 0.001). At follow-up cases no longer used bifocals or progressives as a basic solution, and none of them reported that they solely relied on these visual aids. Instead, many more cases used CCTV at follow-up than at baseline. The control group reported no significant change in use of visual aids.

**Figure 2:**
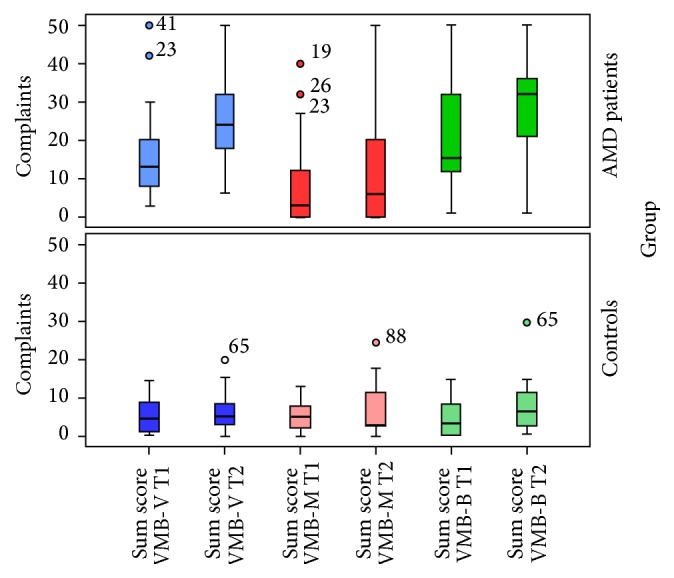
Visual, musculoskeletal, and balance complaints for AMD patients and referents with age-normal vision at baseline and at follow-up 4 years later. Boxplots, showing quartiles and median values, with whiskers showing range and numbered outliers.

Cases reported significantly more visual, musculoskeletal, and balance complaints at follow-up than at baseline, but no change in the level of complaints was found for the referents (Figure 2). Complaints increased among cases from 3 times (VMB-M) to 10 times (VMB-V) as much as among referents. General health decreased in cases, where fewer reported good general health at follow-up than at baseline, whereas there was no significant deterioration of perceived general health found in referents.

### 3.1. Factors Influencing Visual, Musculoskeletal, and Balance Complaints

In regard to the first aim, GEE analysis of complaints showed significant group influences, indicating that the increase of visual, musculoskeletal, and balance complaints from baseline to follow-up was larger in cases than in referents (*B* = 15.177, 95% CI: 11.467; 18.888, *p* < 0.001, *B* = 4.180, 95% CI: 0.018; 8.342, *p* = 0.049, and *B* = 18.862, 95% CI: 14.373; 23.351, *p* < 0.001, resp.).

In support for the second aim, and as shown in Table 3, visual and balance complaints increased in step with deteriorating VA in cases, along with the need for larger critical print size. The analyses also revealed that all three complaints increased with increased use of magnification and deteriorating visual function.

Also in referents, visual complaints increased with declining VA, resulting in more visual and balance complaints as visual function deteriorated (measured by VFQ-NAS), but there were hardly no changes in magnification and thereby no association between magnification and any of the three VMB complaints.

To further evaluate influences from use of visual aids, types of aid were regressed on visual, musculoskeletal, and balance complaints, serving as dependent variables. As shown in Table 3, these analyses showed that in cases reduced use of bifocals and progressives was associated with decreased visual complaints, while increased use of CCTV was associated with increased visual, musculoskeletal, and balance complaints. In referents, the use of reading glasses was associated with decreased visual complaints and use of bifocals was associated with increased visual and balance complaints.

### 3.2. Factors Influencing Perceived General Health

In support for the third aim, GEE analyses showed that cases perceived poorer health with increasing visual and musculoskeletal complaints (Table 4). The need for larger font size was also a marker for poorer perceived health in cases. Referents perceived poorer health in combination with increased visual and balance complaints.

## 4. Discussion

This study followed a group of AMD patients and age-matched referents responding to reported change in perceived complaints during visual decline over a period of four years. All results supported the three hypotheses proposed in this study. That is, AMD patients were more at risk of increased visual, musculoskeletal, and balance complaint than similarly aged individuals with age-normal vision. Additionally, visual deterioration was a risk marker for increased visual and balance complaints both in cases and in referents. In referents this decrease must be considered consistent with the age-normal decline [18]. Finally, increased visual, musculoskeletal, and balance complaints constituted risk factors for decreased perceived general health.

### 4.1. Group Differences

At baseline AMD patients had significantly worse conditions in all assessed areas compared to referents, except for reported musculoskeletal complaints. Most of these group differences remained or increased at follow-up, which supports the hypothesis that having AMD entails a greater risk of poorer health [7–9]. It can be hypothesized that, in pace with visual function decline and increased need for support and assistance, low vision patients may successively abandon physical and social activities resulting in a less satisfactory quality of life [5, 6, 8, 9, 34].

### 4.2. Factors Influencing Visual, Musculoskeletal, and Balance Complaints

Visual deterioration, such as worsening BCVA, need of larger print size, larger magnification when reading, and worsened near visual functioning were risk markers for increased visual and balance complaints among AMD patients. Interestingly, the referents showed a similar pattern to AMD patients, in regard to BCVA and near visual functioning. Thus, visual deterioration seems to be a risk marker for increasing visual and balance complaints regardless of initial vision status (normal or impaired). These results are of course consistent with general deterioration of sensory function with age; however, the increased magnitude of complaints is more profound among AMD patients, which may put them at higher risk of developing lower health and lower quality of life than those with same age but with age-normal vision (Table 3).

Reduced near vision function and increased need of magnification were the most prominent risk markers for musculoskeletal complaints among AMD patients. This result is consistent with previous research showing that during near work tasks an increased use of optical enlargement in visual enhancing devices may adversely result in restricted postures [22, 26, 27, 35] that subsequently can lead to increased musculoskeletal complaints as well as balance complaints [15, 17].

Magnifying aids facilitate everyday life but may have side effects such as limited and restricted field of view and the need to adopt awkward (nonneutral) head and body postures to see as well as possible. As a consequence, magnifying aids may lead to increased strain on the visual and musculoskeletal systems, subsequently resulting in increased complaints. When the portable visual aids are not sufficient any longer, stationary visual aids as CCTV may be needed, on the cost of increasing strain on the visual and musculoskeletal systems. In the present study, the increased use of visual enhancing devices such as CCTV was related to increased visual and musculoskeletal complaints in AMD patients. Thus, as vision deteriorates increased visual inputs from use of specialized visual enhancing aids, such as hyperoculars, magnifiers, and CCTV, are needed, where AMD patients need to adopt certain postures to adjust the eye to the best position for viewing. These adopted postures can be compared in many ways to those found in people using visual display units (VDU) at work, where visual ergonomics are monitored carefully in order to prevent neck/scapular complaints [36–38]. However, visual ergonomic guidelines might be overlooked when providing CCTV, especially as they are situated in old patients' homes (or nursing homes) with limited ability for adequate adjusting. One should, however, bear in mind that CCTV is typically prescribed in situations when near vision is severely impaired and when the enlargement gained by ordinary optical enhancing devices is insufficient. CCTV is thus often one of a very few remaining solutions when extreme magnification is needed.

Normally a two times increase in dioptric power represents three line improvements on the near reading test chart but may not be applicable as the distance between the eye and the magnifier may vary [23]. Our estimates of magnification at use do not intend to estimate angular enlargement but give a hint of the increasing limits of high powered visual aids.

### 4.3. Perceived General Health

At baseline, fewer cases, proportionately, than referents perceived themselves as healthy. At follow-up, the number of cases perceiving themselves as unhealthy had increased, while there was no significant change among referents. Visual function (need for a larger font size), visual complaints, and musculoskeletal complaints constituted risk markers for perceived unhealthiness in AMD patients, and visual and balance complaints were significant risk markers among referents. Thus, aspects of visual loss affect perceived health as people grow older [9], irrespective of whether they have visual impairment. However, the magnitude of complaints is larger among AMD patients, thus increasing the risk of developing lower health. Our results are in line with studies showing that visual functional loss is associated with depression [5–8] and that loss of vision is one of the most-feared disabilities [6].

Increased musculoskeletal complaints were associated with decreased health in cases only. This is in line with findings in a previous study describing decreased health and increased need of health care in people with neck or back pain [39]. Also the association of increased balance complaints and unhealthiness is consistent with previous research [5, 9, 11, 12, 40]. However this association was only noticed among referents. This result was somewhat unexpected since both groups showed increased balance complaints and decreased general health over time. The lack of association found between balance complaints and health in AMD patients may be statistical in nature and due to ceiling effect. That is, because AMD patients already had a greater level of balance problems than referents (by four times) the increase of balance complaints from an already high level may have less effect on perceived health than a similar magnitude of increase from much lower levels, as in the control group. However, this hypothesis should be tested in further research.

### 4.4. Limitations

The overall dropout rate was 38%, with a larger dropout rate in the AMD group. Given that this study was conducted in a group of elderly participants who have an increased risk of illness and mortality, this rate was to be expected. The larger proportion of dropouts due to mortality in AMD patients is consistent with research showing that among the elderly late-stage AMD is associated with increased risk of all-cause mortality [8, 9]. Since the mortality rate is probably larger among those with the most complaints and the poorest health, the effect of this attrition bias would probably decrease any differences between AMD patients and referents; thus, differences between the groups may be larger than those reported here and observed differences are probably underrated.

It should be noted that six AMD patients had ranibizumab injections at the low vision clinic prior to registration and two participants (one AMD patient and one referent) had been treated with monocular cataract surgery between registration and follow-up. This may have slowed down their visual deterioration and thus any potential influences on visual, musculoskeletal, or balance complaints in the AMD group. Therefore, the observed level of complaints may underestimate the level in an AMD population not being treated with eye surgery or receiving ranibizumab injections.

We hypothesized that magnifying visual aids may give rise to suboptimal ergonomic head postures that affect the muscles in the neck scapular area, resulting in subsequent musculoskeletal complaints and increasing the risk of more balance complaints. At the same time, we cannot neglect the fact that in late-stage AMD visual performance may not be sufficient for adequate visuomotor support. Research in this area is limited and future research is warranted.

It should be noted that our estimates of magnification do not reflect the exact equivalent viewing power (EVP) as we did not collect all required distances for this estimate, that is, the distances between the naked eye/reading glass and the magnifying aid compared to EVP. The magnification estimate can therefore be somewhat overestimated. However, the magnification estimate reflects the use of higher dioptric power under conditions of deteriorating visual function. In pace with need of higher amounts of magnification, the reading distance variation gets more limited and the focal depth gets more specific resulting in the need for adopting a more constricted and static posture [22, 26, 28].

It was not possible to separate the influences of visual aids from the influences of loss of central vision in the present data because these factors are intertwined. That is, AMD patients start increasing the use of visual aids when they start losing their central vision. Future research measuring the amount of time in visual aid use might shed light on the magnitude of influence from visual aids and from loss of central vision

This study had a longitudinal design where temporal order of causality was met. However, the result that visual decline evoked increased visual, musculoskeletal, and balance complaints does not reveal the mechanisms behind these findings or whether there are other variables involved in this process, such as personality, depression, or socioeconomic status. Prevalence and intervention studies could shed light on how best to prevent increased complaints in people with AMD and on treatments to decrease complaints among those affected. Thus future research may shed light on moderating and mediating variables in this process.

## 5. Conclusion

The present study demonstrated that visual, musculoskeletal, and balance complaints increase with visual decline and increase more among people with AMD than among people with normal vision. Increased visual, musculoskeletal, and balance complaints were also found to negatively affect general health. The study showed that visual decline and increased use of greater magnification in visual aids are important risk factors for increased complaints.

### 5.1. Clinical Implications

The results from this study show that AMD patients' use of magnifying visual aids has side effects that optometrists and low vision staff must be aware of when prescribing visual aids. As a preventive measure, optometrists may suggest alternative use of both optical and technical visual enhancing aids as well as using devices with* text-to-speech* function (e.g., electronic readers) when applicable. Visual aids are important, especially in elderly people with severe AMD, as these aids enable users to continue daily activities and maintain their quality of life. Optometrist may also refer patients to a physiotherapist for investigation, treatment, and preventive training [40–42]. This calls for coordinated actions at an early stage in order to prevent visual-related musculoskeletal and balance complaints in AMD patients to minimize the further risk of more serious complaints.

## Figures and Tables

**Table 1:** Descriptive statistics of AMD patients and reference group at baseline.

	AMD patients (*n* = 64)	Control group (*n* = 24)	Group difference	*p* ^*∗*^
	*M* (SD)	*M* (SD)		

BCVA (logMAR)	0.78 (0.38)	−0.03 (0.09)	0.81	<0.001
Critical print size (p)	7.31 (5.01)	4.79 (0.51)	2.52	0.005
Reading distance (cm)	20.27 (7.82)	41.10 (6.87)	−20.83	<0.001
Magnification (×)	4.12 (2.52)	0.62 (0.07)	3.50	<0.001
VFQ-NAS (0–100)	42.24 (19.69)	97.05 (3.78)	−54.81	<0.001
VMB-V (0–50)	15.79 (11.09)	4.81 (3.89)	10.98	<0.001
VMB-M (0–50)	8.07 (10.29)	5.98 (5.95)	2.09	0.867
VMB-B (0–50)	20.80 (12.94)	4.92 (4.21)	15.88	<0.001
Healthy, *n* (%)	36 (56)	19 (79)	−23%	0.049

	*n* (%)	*n* (%)		

*Type of near visual aid*				
Reading glasses, *n* (%)	10 (16)	6 (25)	−9%	0.357
Bifocals, *n* (%)	9 (14)	6 (25)	−11%	0.339
Progressives, *n* (%)	4 (6)	12 (50)	−44%	<0.001
Handheld aids, *n* (%)	3 (5)	0 (0)	5%	0.559
CCTV, *n* (%)	5 (8)	0 (0)	8%	0.317
Combination^†^, *n* (%)	33 (52)	0 (0)	52%	<0.001

^*∗*^ *p* values according to Mann–Whitney *U* test. ^†^Any combination of two or more aids. BCVA: best corrected visual acuity; VFQ-NAS: Visual Functioning Questionnaire-Near Activities Subscale; VMB: visual, muscular, and balance complaints questionnaire.

**Table 2:** Descriptive statistics of AMD patients and age-matched control group with normal vision at baseline and follow-up.

	Baseline	Follow-up	Difference	*p* ^*∗*^
*Cases (n* = 37)				
BCVA (logMAR)	0.78	0.91	0.19	<0.001
Critical print size (p)	6.89	25.78	18.89	<0.001
Magnification (×)	3.74	5.64	1.91	0.004
Reading distance (cm)	20.11	19.28	−0.83	0.436
VFQ-NAS	45.72	31.45	−4.26	<0.001
VMB-V	15.19	26.32	11.13	<0.001
VMB-M	8.03	11.99	3.96	0.019
VMB-B	20.99	28.64	7.65	<0.001
Healthy, *n* (%)	27 (73)	18 (49)	−9 (33)	0.007
*Type of visual aid*				
Reading glasses, *n* (%)	7 (19)	3 (8)	−4 (43)	0.157
Bifocals, *n* (%)	7 (19)	0 (0)	−7 (100)	0.008
Progressives, *n* (%)	4 (11)	0 (0)	−4 (100)	0.046
Handheld aids, *n* (%)	0 (0)	0 (0)	0 (0)	1.000
CCTV, *n* (%)	3 (8)	19 (51)	16 (633)	<0.001
Combination^†^, *n* (%)	16 (43)	15 (41)	−1 (6)	0.819

*Referents (n* = 18)				
BCVA (logMAR)	−0.03	0.03	0.06	0.030
Critical print size (p)	4.76	4.17	−0.50	0.070
Magnification (×)	0.61	0.62	0.01	0.588
Reading distance (cm)	40.91	36.44	−4.47	0.026
VFQ-NAS	96.99	91.61	−5.37	0.010
VMB-V	4.92	6.33	1.41	0.200
VMB-M	5.06	6.14	1.08	0.477
VMB-B	4.42	7.36	2.94	0.102
Healthy, *n* (%)	13 (72)	12 (67)	−1 (8)	0.655
*Type of visual aid*				
Reading glasses, *n* (%)	4 (22)	3 (17)	−1 (25)	0.564
Bifocals, *n* (%)	5 (28)	3 (17)	−2 (40)	0.157
Progressives, *n* (%)	9 (50)	12 (67)	3 (133)	0.180
Handheld aids, *n* (%)	0 (0)	0 (0)	0 (0)	1.000
CCTV, *n* (%)	0 (0)	0 (0)	0 (0)	1.000
Combination^†^, *n* (%)	0 (0)	0 (0)	0 (0)	1.000

^*∗*^ *p* values according to Wilcoxon signed rank test. ^†^Any combination of two or more aids. BCVA: best corrected visual acuity; VFQ-NAS: Visual Function Questionnaire-Near Activities Subscale; VMB: visual, musculoskeletal, and balance complaints questionnaire.

**Table 3:** Univariate generalized estimating equations on visual-related variable change from baseline to follow-up regressed on visual, musculoskeletal, and balance complaints in AMD patients and age-matched referents.

Independent variables	Visual complaints			Musculoskeletal complaints			Balance complaints		
	*B*	95% CI	*p*	*B*	95% CI	*p*	*B*	95% CI	*p*
*Cases (n* = 37)									
BCVA (logMAR)	**0.56**	**0.20; 0.93**	**0.002**	0.31	−.032; 0.94	0.338	**0.54**	**0.31; 0.77**	**<0.001**
Critical print size (p)	**0.01**	**0.01; 0.01**	**<0.001**	0.01	−0.00; 0.01	0.255	**0.01**	**0.00; 0.01**	**<0.001**
Reading dist. (cm)	−0.01	−0.02; 0.01	0.530	0.03	−0.01; 0.06	0.190	0.00	−0.02; 0.02	0.852
Magnification (×)	**0.06**	**0.02; 0.11**	**0.003**	**0.08**	**0.01; 0.15**	**0.034**	**0.06**	**0.03; 0.09**	**<0.001**
VFQ-NAS (0–100)	**−0.02**	**−0.03; −0.01**	**<0.001**	**−0.02**	**−0.03; −0.01**	**0.031**	**−0.01**	**−0.02; −0.01**	**<0.001**
*Vision aids*									
Reading glasses	−0.22	−0.58; 0.14	0.234	−0.60	−1.34; 0.14	0.111	−0.24	−0.53; 0.05	0.110
Bifocals	**−0.87**	**−1.15; −0.58**	**<0.001**	−0.52	−1.33; 0.29	0.205	−0.13	0.46; 0.21	0.462
Progressives	**−0.63**	**−1.20; −0.07**	**0.027**	−1.52	−3.78; 0.74	0.187	0.05	−0.20; 0.30	0.705
CCTV	**0.54**	**0.39; 0.68**	**<0.001**	**0.38**	**0.02; 0.74**	**0.039**	**0.35**	**0.17; 0.53**	**<0.001**
Combination^*∗*^	−0.10	0.35; −0.16	0.464	0.16	−0.19; 0.51	0.374	**−0.20**	**−0.40; 0.01**	**0.037**

*Referents (n* = 18)									
BCVA (logMAR)	**1.99**	**0.02; 3.95**	**0.047**	1.51	−0.34; 3.36	0.110	2.14	−0.67; 4.94	0.135
Critical print size (p)	−0.02	−0.17; 0.12	0.739	0.14	−0.27; 0.56	0.504	0.06	−0.31; 0.43	0.750
Reading dist. (cm)	−0.02	−0.05; 0.02	0.267	−0.01	−0.05; 0.03	0.680	−0.05	−0.12; 0.02	0.194
Magnification (×)	0.88	−0.16; 1.91	0.096	0.85	−0.99; 2.70	0.366	1.47	−0.63; 3.57	0.171
VFQ-NAS (0–100)	**−0.02**	**−0.05; 0.00**	**0.028**	−0.02	−0.05; 0.01	0.269	**−0.04**	**−0.06; −0.01**	**0.005**
*Vision aids*									
Reading glasses	**−0.65**	**−0.85; −0.46**	**<0.001**	−0.11	−0.69; 0.48	0.725	−0.10	−0.60; 0.39	0.681
Bifocals	**0.66**	**0.22; 1.10**	**0.003**	0.34	−0.27; 0.96	0.273	**0.77**	**0.16; 1.35**	**0.013**
Progressives	−0.01	−0.36; 0.34	0.966	−0.15	−0.65; 0.34	0.560	−0.52	−1.11; 0.07	0.084
CCTV	—			—			—		
Combination^*∗*^	—			—			—		

^*∗*^Any combination of two or more aids. BCVA: best corrected visual acuity; VFQ-NAS: Visual Function Questionnaire-Near Activities Subscale; VMB: visual, musculoskeletal, and balance complaints questionnaire. GEE model specifications: unstructured correlation matrix, normal distribution, and log link function.

**Table 4:** Univariate GEE logistic analysis odds ratio for perceived health compared with perceived poor health among AMD patients and age-matched referents with normal vision.

Independent variables	OR	95% CI	*p*
*Cases (n* = 37)			
BCVA (logMAR)	0.58	0.15–2.24	0.427
Critical print size (p)	0.98	0.96–0.99	0.011
Reading dist. (cm)	0.98	0.93–1.04	0.536
Magnification (×)	0.95	0.83–1.09	0.444
VFQ-NAS (0–100)	1.02	0.99–1.05	0.140
VMB-V	0.97	0.94–1.00	0.050
VMB-M	0.95	0.91–1.00	0.041
VMB-B	0.98	0.95–1.01	0.166

*Referents (n* = 18)			
BCVA (logMAR)	0.03	0.00–5.43	0.179
Critical print size (p)	0.53	0.18–1.53	0.239
Reading distance (cm)	0.99	0.93–1.05	0.620
Magnification (×)	0.18	0.00–132.31	0.607
VFQ-NAS (0–100)	1.11	0.99–1.24	0.066
VMB-V	0.67	0.51–0.88	0.004
VMB-M	0.90	0.76–1.07	0.232
VMB-B	0.74	0.61–0.90	0.003

BCVA: best corrected visual acuity; VFQ-NAS: Visual Function Questionnaire-Near Activities Subscale; VMB: visual, musculoskeletal, and balance questionnaire.

## References

[1] Ferris F. L., Wilkinson C. P., Bird A. (2013). Clinical classification of age-related macular degeneration. *Ophthalmology*.

[2] Jager R. D., Mieler W. F., Miller J. W. (2008). Age-related macular degeneration. *The New England Journal of Medicine*.

[3] Hyman L., Neborsky R. (2002). Risk factors for age-related macular degeneration: an update. *Current Opinion in Ophthalmology*.

[4] Friedman D. S., O'Colmain B. J., Muñoz B. (2004). Prevalence of age-related macular degeneration in the United States. *JAMA Ophthalmology*.

[5] Brody B. L., Gamst A. C., Williams R. A. (2001). Depression, visual acuity, comorbidity, and disability associated with age-related macular degeneration. *Ophthalmology*.

[6] Dev M. K., Paudel N., Joshi N. D., Shah D. N., Subba S. (2014). Impact of visual impairment on vision-specific quality of life among older adults living in nursing home. *Current Eye Research*.

[7] Slakter J. S., Stur M. (2005). Quality of life in patients with age-related macular degeneration: impact of the condition and benefits of treatment. *Survey of Ophthalmology*.

[8] Fisher D. E., Jonasson F., Eiriksdottir G. (2015). Age-related macular degeneration and mortality in community-dwelling elders: the age, gene/environment susceptibility Reykjavik study. *Ophthalmology*.

[9] McCarty C. A., Nanjan M. B., Taylor H. R. (2001). Vision impairment predicts 5 year mortality. *British Journal of Ophthalmology*.

[10] Lord S. R. (2006). Visual risk factors for falls in older people. *Age and Ageing*.

[11] Lee H. K. M., Scudds R. J. (2003). Comparison of balance in older people with and without visual impairment. *Age and Ageing*.

[12] Ray C. T., Horvat M., Croce R., Mason R. C., Wolf S. L. (2008). The impact of vision loss on postural stability and balance strategies in individuals with profound vision loss. *Gait & Posture*.

[13] Willis J. R., Vitale S. E., Agrawal Y., Ramulu P. Y. (2013). Visual impairment, uncorrected refractive error, and objectively measured balance in the united states. *JAMA Ophthalmology*.

[14] Salonen L., Kivelä S.-L. (2012). Eye diseases and impaired vision as possible risk factors for recurrent falls in the aged: a systematic review. *Current Gerontology and Geriatrics Research*.

[15] McPartland J. M., Brodeur R. R., Hallgren R. C. (1997). Chronic neck pain, standing balance, and suboccipital muscle atrophy—a pilot study. *Journal of Manipulative and Physiological Therapeutics*.

[16] Naz I., Yildirim Y. (2010). Investigation of the relationship between wearing glasses and deep cervical flexor endurance in patients with non-specific neck pain. *Journal of Back and Musculoskeletal Rehabilitation*.

[17] Yahia A., Ghroubi S., Jribi S. (2009). Chronic neck pain and vertigo: is a true balance disorder present?. *Annals of Physical and Rehabilitation Medicine*.

[18] Dagnelie G. (2013). Age-related psychophysical changes and low vision. *Investigative Ophthalmology & Visual Science*.

[19] Roos P. E., Dingwell J. B. (2013). Using dynamic walking models to identify factors that contribute to increased risk of falling in older adults. *Human Movement Science*.

[20] Zetterlund C., Lundqvist L.-O., Richter H. O. (2009). The relationship between low vision and musculoskeletal complaints. a case control study between age-related macular degeneration patients and age-matched controls with normal vision. *Journal of Optometry*.

[21] Mitchell P., Wang J. J., Foran S., Smith W. (2002). Five-year incidence of age-related maculopathy lesions: the Blue Mountains Eye Study. *Ophthalmology*.

[22] Rosenberg E. A., Sperazza L. C. (2008). The visually impaired patient. *American Family Physician*.

[23] Johnston A. W. (2003). Understanding how simple magnifiers provide image enlargement. *Clinical and Experimental Optometry*.

[24] Mon-Williams M., Burgess-Limerick R., Plooy A., Wann J. (1999). Vertical gaze direction and postural adjustment: an extension of the Heuer Model. *Journal of Experimental Psychology: Applied*.

[25] Richter H. O. (2014). Neck pain brought into focus. *Work*.

[26] Krueger H., Conrady P., Zulch J. (1989). Work with magnifying glasses. *Ergonomics*.

[27] Macnaughton J. (2009). *Eye Essentials: Low Vision Assessment*.

[28] Latham K., Tabrett D. R. (2012). Guidelines for predicting performance with low vision aids. *Optometry & Vision Science*.

[29] Lovie-Kitchin J. (2011). Reading with low vision: the impact of research on clinical management. *Clinical and Experimental Optometry*.

[30] Kodjebacheva G., Coleman A. L., Ensrud K. E. (2010). Reliability and validity of abbreviated surveys derived from the National Eye Institute Visual Function Questionnaire: the study of osteoporotic fractures. *American Journal of Ophthalmology*.

[31] Revicki D. A., Rentz A. M., Harnam N., Thomas V. S., Lanzetta P. (2010). Reliability and Validity of the National Eye Institute Visual Function Questionnaire-25 in patients with age-related macular degeneration. *Investigative Ophthalmology and Visual Science*.

[32] Lundqvist L.-O., Zetterlund C., Richter H. O. (2016). Reliability and validity of the visual, musculoskeletal, and balance complaints questionnaire. *Optometry Vision Science*.

[33] Twisk J. W. R. (2004). Longitudinal data analysis. A comparison between generalized estimating equations and random coefficient analysis. *European Journal of Epidemiology*.

[34] Lee E.-K. O., Brennan M. (2006). Stress constellations and coping styles of older adults with age-related visual impairment. *Health and Social Work*.

[35] Bowers A., Cheong A. M. Y., Lovie-Kitchin J. E. (2007). Reading with optical magnifiers: page navigation strategies and difficulties. *Optometry and Vision Science*.

[36] Anshel J. R. (2007). Visual ergonomics in the workplace. *AAOHN Journal*.

[37] Long J., Richter H. (2014). Visual ergonomics at work and leisure. *Work*.

[38] Richter H. O., Crenshaw A. G., Lyskov E. (2007). Accomodation—vergence performance after low levels of oculomotor load. *Scandinavian Journal of Work, Environment and Health, Supplement*.

[39] Woodhouse A., Pape K., Romundstad P. R., Vasseljen O. (2016). Health care contact following a new incident neck or low back pain episode in the general population; the HUNT study. *BMC Health Services Research*.

[40] Radvay X., Duhoux S., Koenig-Supiot F., Vital-Durand F. (2007). Balance training and visual rehabilitation of age-related macular degeneration patients. *Journal of Vestibular Research: Equilibrium and Orientation*.

[41] Holmberg C., Rappenecker J., Karner J. J., Witt C. M. (2014). The perspectives of older women with chronic neck pain on perceived effects of qigong and exercise therapy on aging: a qualitative interview study. *Clinical Interventions in Aging*.

[42] Lundqvist L.-O., Zetterlund C., Richter H. O. (2014). Effects of feldenkrais method on chronic neck/scapular pain in people with visual impairment: a randomized controlled trial with one-year follow-up. *Archives of Physical Medicine and Rehabilitation*.

